# Supplementary material for: Introducing dorsoventral patterning in adult regenerating lizard tails with gene-edited embryonic neural stem cells
Source: Nat Commun. 2021 Oct 14;12:6010. doi: 10.1038/s41467-021-26321-9 (PMC8516916; doi:10.1038/s41467-021-26321-9)
Supplement: Supplementary file 1 — Supplementary Information [file 41467_2021_26321_MOESM1_ESM.pdf]

Supplementary Information for

Introducing dorsoventral patterning into adult regenerating lizard tails with gene-edited embryonic neural stem cells

Thomas P. Lozito, Ricardo Londono, Aaron X. Sun, Megan L. Hudnall

Thomas P. Lozito
Email: lozito@usc.edu

This PDF file includes:

Supplementary Figures 1 to 21
Supplementary Table 1
Supplementary References

Supplementary Figure 1: Comparisons of embryonic lizard tail development and adult tail regeneration. (A-C) Light microscopy of lizard embryos 2, 7, and 14 days post-oviposition (DPO) highlighting tail development. **(A, Inset)** Cross section of a lizard embryo tail bud analyzed by Sox2 immunofluorescence (IF) and Edu staining. **(D-F)** Micrographs of regenerating lizard tails 14, 21, and 28 days post-amputation (DPA). (D, Inset) Fluorescence micrograph of a blastema cross section analyzed by Sox2 IF and EdU staining. Dashed lines denote amputation planes. et, ependymal tube; nt neural tube. Bar = 0.5 mm for Panels A-C. Bar = 5 mm for Panels D-F. Bar = 200 μm for A and D inset.

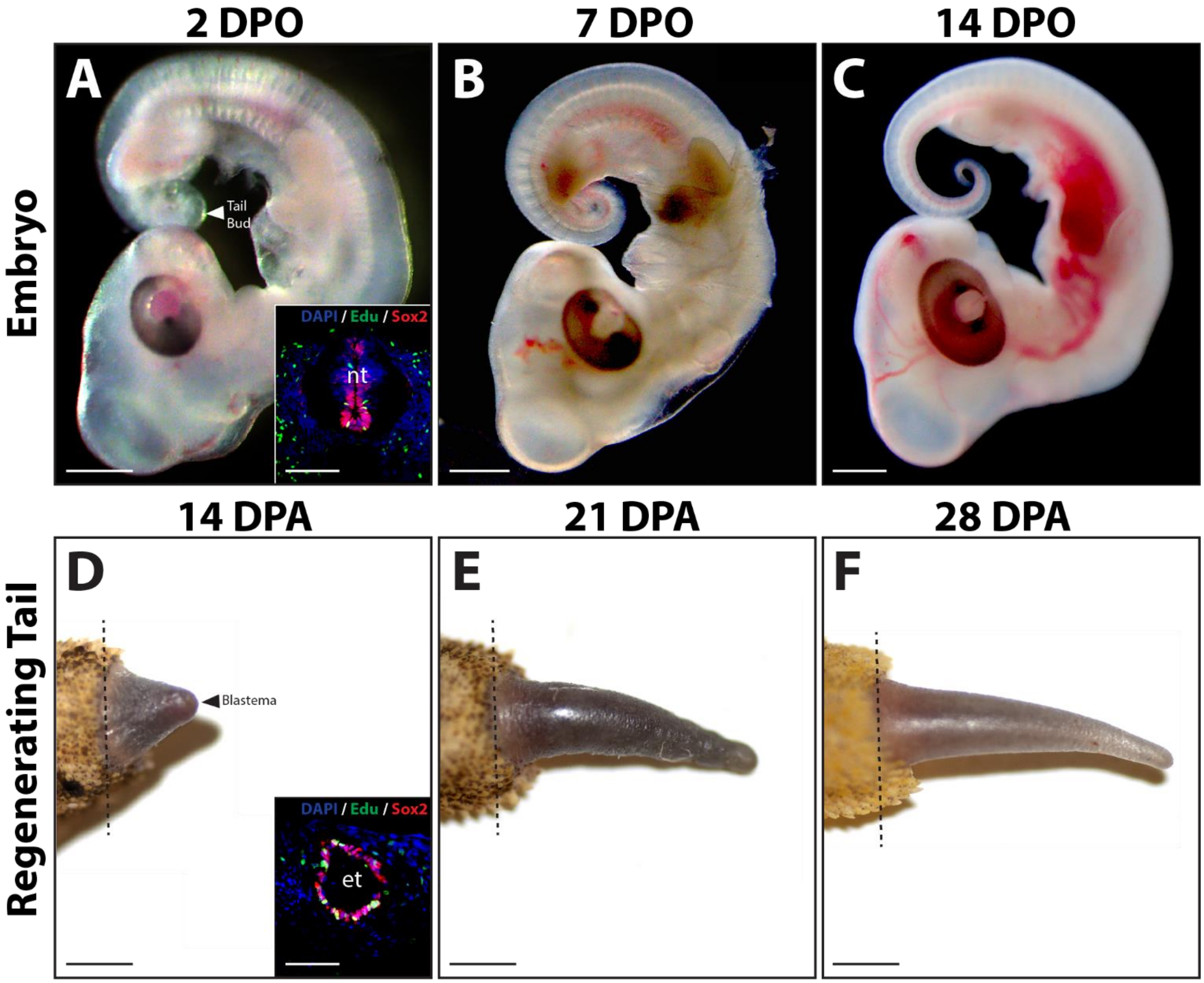

Supplementary Figure 2: Quantification of embryonic and adult NSC neurosphere dorsoventral identities. NSCs were isolated from either embryonic tail NTs or adult tail spinal cords, cultured as neurospheres for 14 days, and co-immunostained for roof plate marker Pax7, lateral domain Pax6, and floor plate marker Shh. Positive signals for each marker were counted among 250 embryonic neurospheres and 250 adult neurospheres and are depicted in the histograms below.

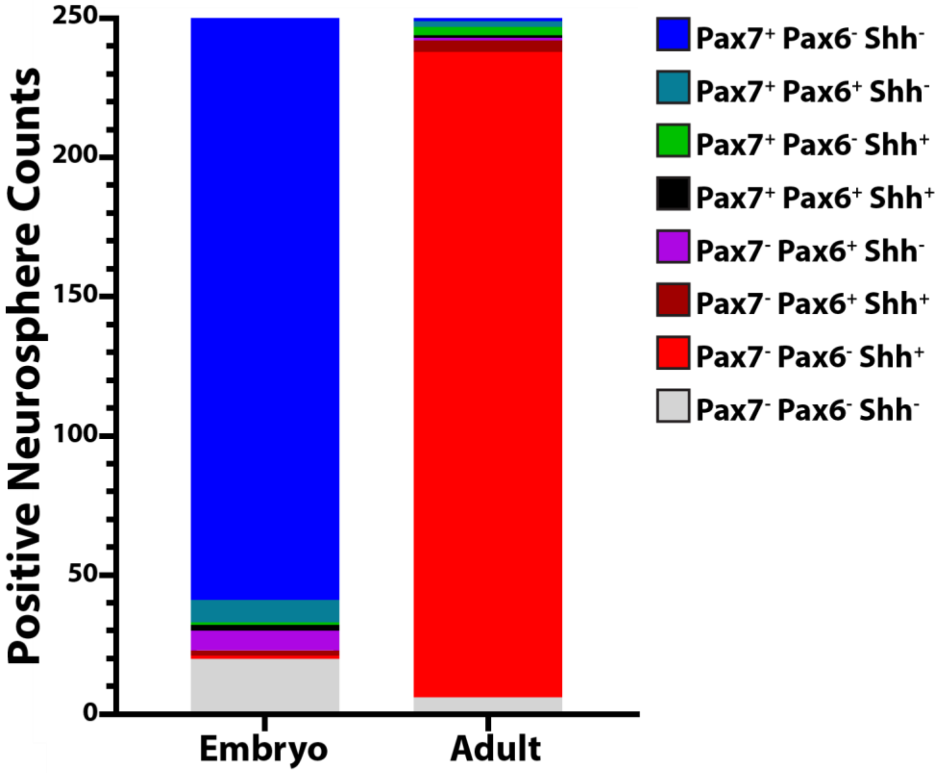

Supplementary Figure 3: Quantification of effects of cyclopamine and SAG on embryonic and adult NSC neurosphere dorsoventral identities. Neurospheres formed from embryonic or adult lizard tail NSCs were treated with 5 μM cyclopamine or 100 nM SAG. NSCs were isolated from either embryonic tail NTs or adult tail spinal cords, cultured as neurospheres for 14 days, and co-immunostained for Pax7, Pax6, and Shh. Positive signals for each marker were counted among 250 embryonic neurospheres and 250 adult neurospheres for each condition (Vehicle control, cyclopamine, and SAG) and are depicted in the histograms below.

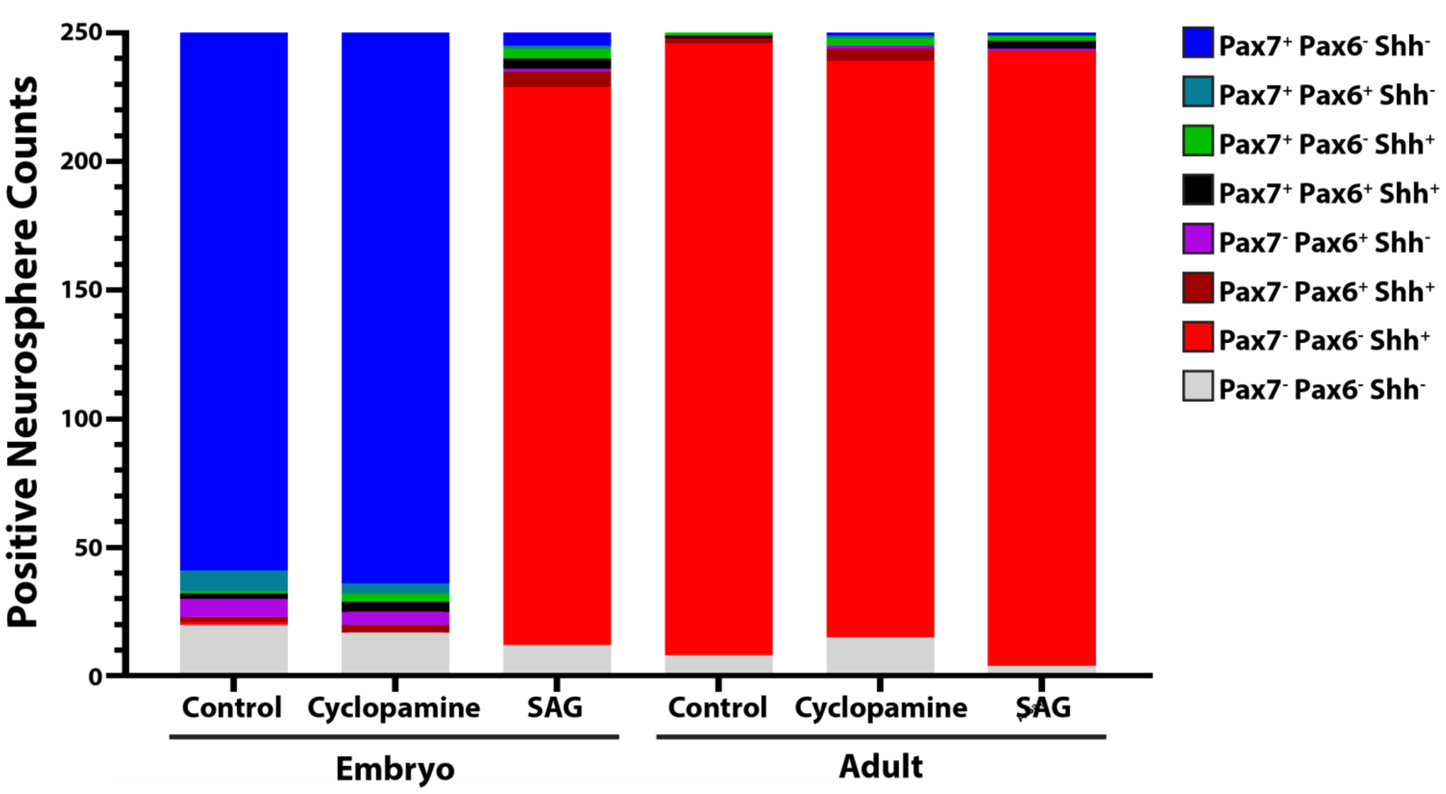

Supplementary Figure 4: NeuN expression among neurons and NSCs in developing and regenerating lizard tails. Cross sections of **(A)** embryonic (14 DPO), **(B)** original (28 DPH), and **(C)** regenerated (28 DPA) tails were analyzed by NeuN, Sox2, and Tuj1 IF. Fluorescence exposure levels for each signal channel are consistent between samples. NeuN co-localized with $Sox2^+$ NSCs in embryonic tails only and with $Tuj1^+$ neurons in adult spinal cords. NeuN was not detected in regenerated tail ETs. et, ependymal tube; nt, neural tube; sce, spinal cord ependyma. Bar = 50 µm.

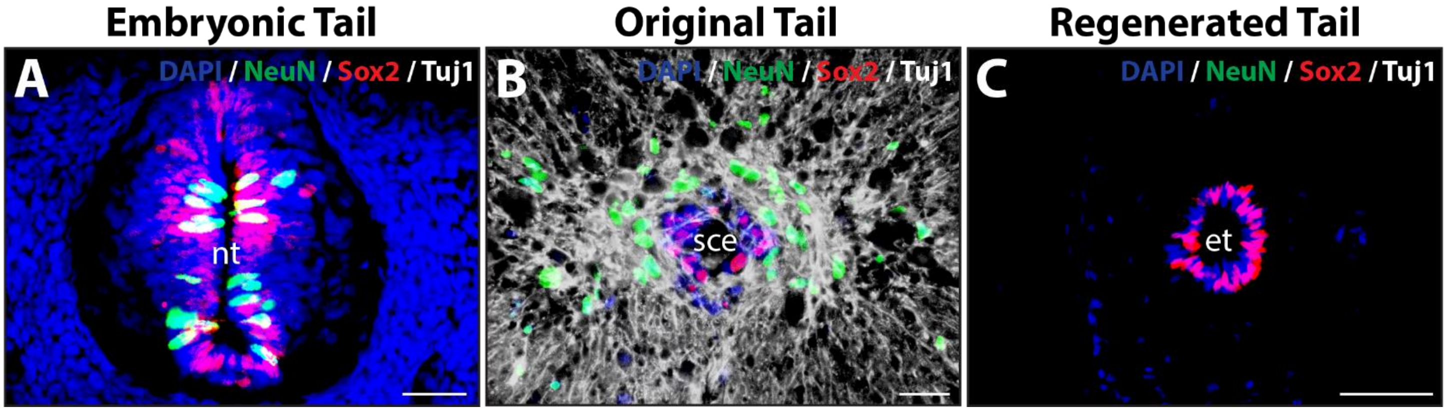

Supplementary Figure 5: Tracing the persistence of insert expression products following viral transduction of live lizard embryos. Freshly laid (0 DP0) lizard eggs were injected with AAV6 particles containing Sox2-Cre inserts and lentiviral particles containing CreStoplight inserts. Tail tissues were collected from embryos (0, 7, and 14 DPO), hatched lizards with original tails (28 and 42 DPH), and hatched lizards with regenerated tails (28 and 42 DPA) and analyzed by Western blots utilizing anti-Cre, -GFP, -RFP, and -Sox2 primary antibodies. Lanes containing molecular weight ladders were increased in contrast until bands were visible and are presented to the left of each blot. Commercially available Sox2 positive control (Abcam) is included in anti-Sox2 blots. Cre was detected in 7 DPO samples but did not persist into later time points, indicating transient expression of AAV-packaged inserts. Conversely, GFP and RFP were detected among embryonic and post-hatching tail tissues, including tissues collected from regenerated tails, indicating permanent integration and continual expression of lentiviral CreStoplight inserts. Sox2 Western blots were included as loading controls since the Sox2 promoter drove expression of Cre in Sox2-Cre constructs and the conversion of GFP to RFP expression in CreStoplight constructs. Source data are provided as a Source Data file.

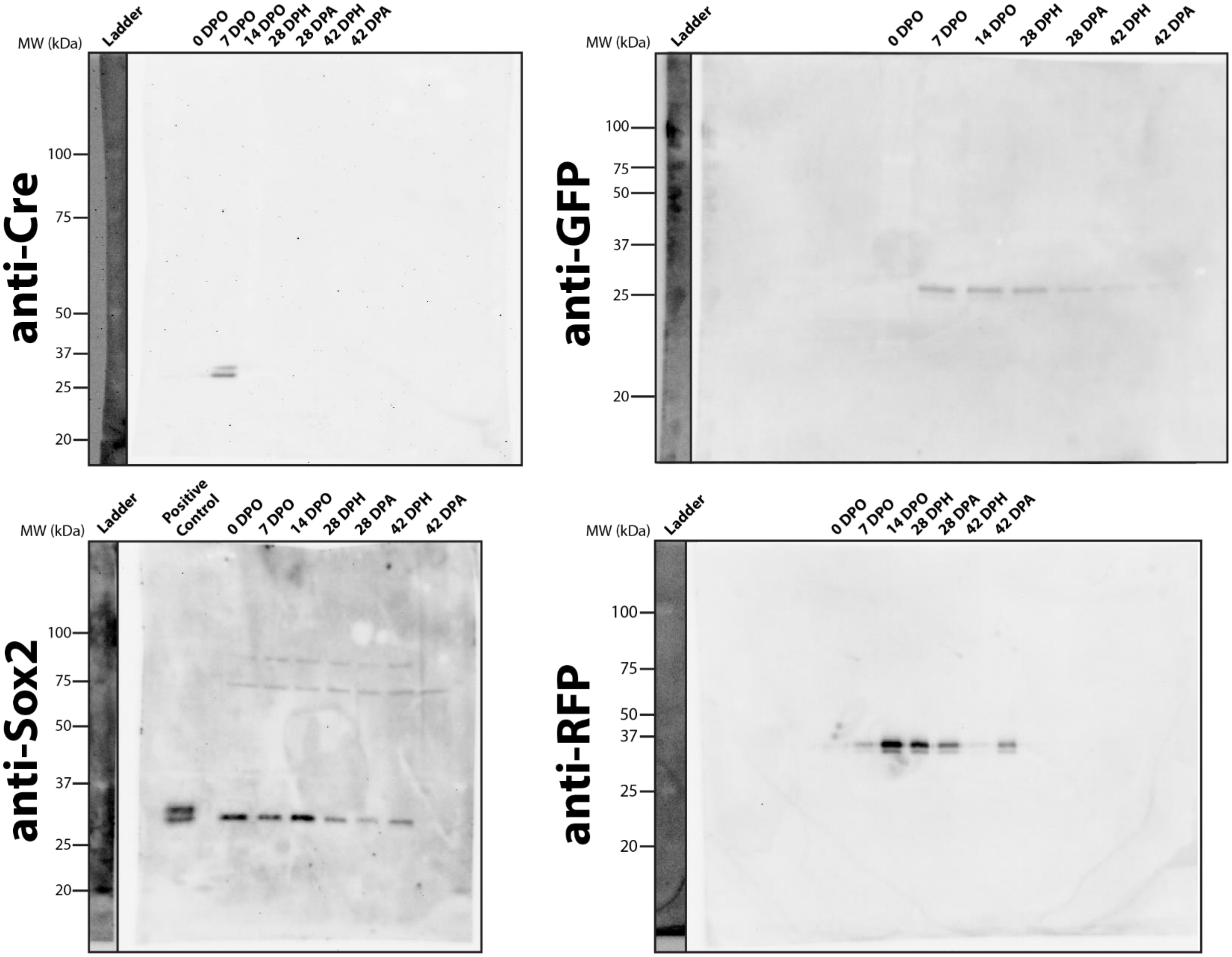

Supplementary Figure 6: Microinjection system for introducing solutions into *L. lugubris* eggs without significantly compromising hatchability rates. Special housing encourages *L. lugubris* lizards to deposit their eggs in specific locations and orientations for optimal accessibility of the embryo for injection. *L. lugubris* is an “egg-gluing” species that adheres their eggs to surfaces, typically under tree bark. Once attached, eggs are nearly impossible to move without damage. Lizard cages were designed and fabricated with removable tops that contain grooves along the edges that simulate lizards’ natural egg laying sites. Approximately 95% of eggs laid by lizards are deposited in these grooves, which are designed with edge heights that facilitate needle insertion at the position of embryos within eggs. Lids containing freshly laid eggs are collected and loaded into a custom-made microinjection system outfitted with a fiber-optic egg candler that allows for visualization of lizard embryos in ovo during controlled injections of up to 20 µl of solutions. Injected eggs are immediately sealed with surgical glue. Eggs injected with PBS using this system exhibit a 99% hatchability rate compared to un-injected controls.

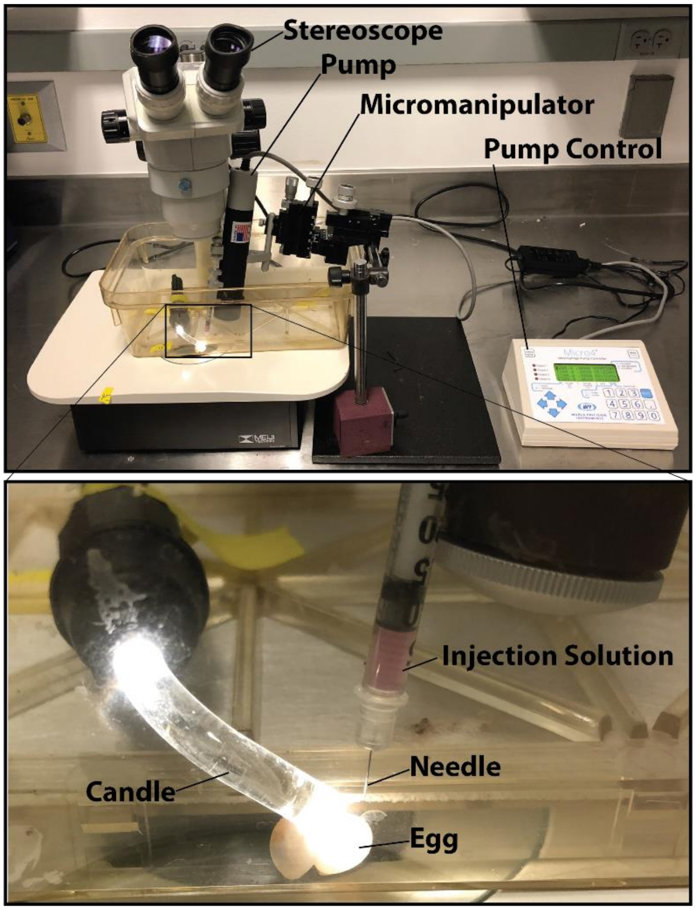

Supplementary Figure 7: Validation of in ovo lentiviral transduction of lizard embryos with CreStoplight constructs. Lentiviral vectors containing CreStoplight inserts were injected into the amniotic sacs of freshly laid (0 DPO) lizard eggs. **(A)** At 7 DPO, green fluorescence was detected throughout lizard embryos. **(B)** Higher magnification view of posterior tissues showing punctate GFP signal by transduced cells. Bar = 50 µm.

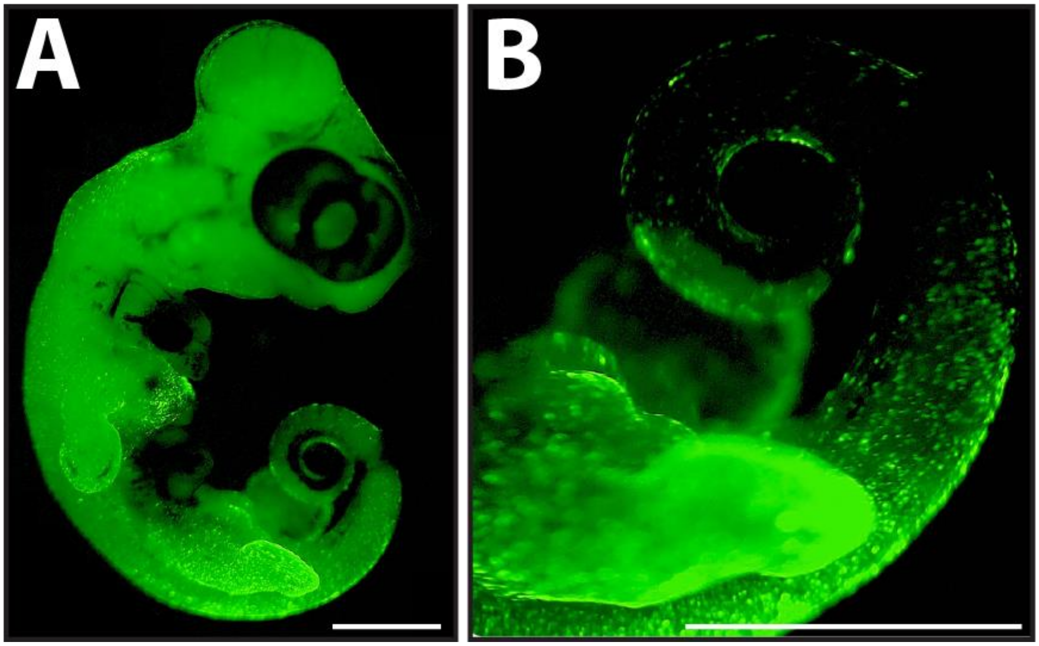

Supplementary Figure 8: Experimental scheme for tracing the differentiation capacity and lineage contributions of Sox2^{+} NT cells *in vivo*. 40 freshly laid (0 DPO) lizard embryos were injected with lentiviral and AAV6 vectors containing CreStoplight and Sox2-Cre constructs, respectively. This system permanently and specifically labeled Sox2^{+} NT cells and their progeny with RFP fluorescence. The differentiation and contribution of RFP-labeled cells to various tail tissues were traced in embryonic lizards 7 and 14 DPO, as well as in post-hatching lizards (28 DPH) and in lizards with regenerated tails (28 DPA). Each time point involved 10 different animals/tails.

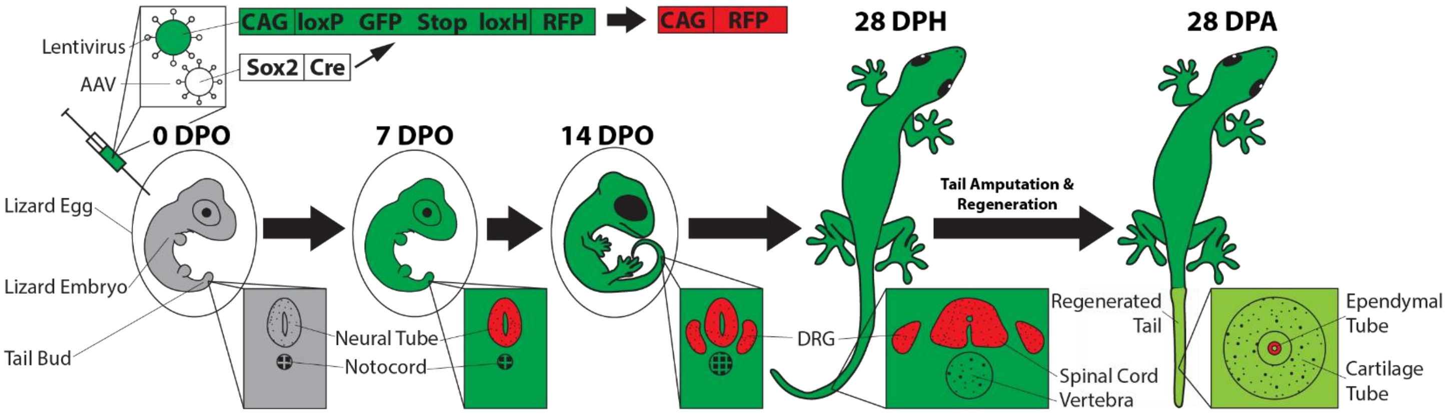

Supplementary Figure 9: Embryonic lizard NT cells contribute to nerve, glia, and NSC populations within developing embryonic tails. Cross sections of **(A-G)** 7 DPO and **(H-N)** 14 DPO tail samples were analyzed for GFP and RFP fluorescence and by Sox2 and Tuj1 IF. At 7 DPO, GFP was expressed by various embryonic tissues, but RFP signal was restricted to cells associated with NTs. The majority of RFP signal was detected in Sox2$^+$ cells, validating effective and specific labeling by the Sox2-Cre/CreStoplight lentiviral system. RFP co-localized with Pax7$^+$, Pax6$^+$, and Shh$^+$ regions of NTs, indicating labeling of roof, lateral, and floor domains, respectively. RFP signal was also detected in Tuj1$^+$ Sox2$^-$ neurons immediately adjacent to NTs, indicating early neurogenesis in Sox2$^+$ NSCs between the time of egg injection and sample collection. At 14 DPO, RFP signal was detected in Sox2$^+$ NSCs and Tuj1$^+$ neurons of NTs. Within NTs, RFP signal continued to be distributed among Pax7$^+$ roof pates, Pax6$^+$ lateral domains, and Shh$^+$ floor plates. DRG had also developed by 14 DPO, and RFP signal co-localized with Tuj1$^+$ DRG regions, indicating derivation of DRG neurons from Sox2$^+$ NT NSCs. Individual signal channels are presented to the left, and merged images of select channels are presented on the right to highlight specific co-expression patterns. White arrow heads denote cells that co-express RFP and Sox2, light grey arrow heads label RFP$^+$ Tuj1$^+$ NT neurons, and dark grey arrow heads mark RFP$^+$ Tuj1$^+$ DRG neurons. drg, dorsal root ganglion; nc, notochord; nt, neural tube. Bar = 50 μm.

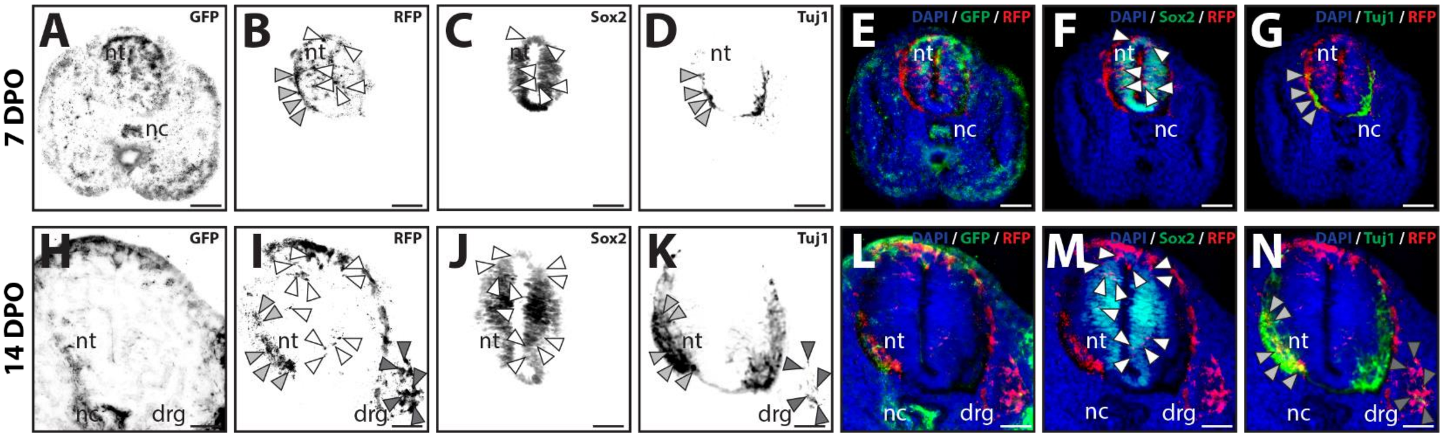

Supplementary Figure 10: Embryonic lizard NT cells contribute to nerve, glia, and NSC populations within adult and regenerated tails. Cross sections of **(A-L)** 28 DPH original tails and **(M-U)** 28 DPA regenerated tails were analyzed for GFP, RFP, Sox2, Tuj1, and GFAP expression. **(F-L)** Higher magnification views of tail spinal cord regions of 28 DPH lizards analyzed in Panels A-E. **(M-U Insets)** lower magnifications of examined regenerated tail ET regions. Original tail samples exhibited RFP signal exclusively in spinal cord and DRG tissues of the CNS. RFP signal co-localized with $Tuj1^{+}$ neurons, $GFAP^{+}$ glial cells, and $Sox2^{+}$ ependymal NSC populations of the spinal cord, as well as $Tuj1^{+}$ neurons of DRG. $Sox2^{+}$ ependymal cells co-expressed Sox2 and GFP, validating the glial/ependymal identity of original tail spinal cord NSCs. RFP signal co-localized with Shh^{+} ependyma regions. In regenerated tails, RFP signal was confined to ETs in glial/ependymal cells that co-expressed Sox2 and GFAP. Individual signal channels are presented to the left, and merged images of select channels are presented on the right to highlight specific co-expression patterns. White arrow heads denote cells that co-express RFP and Sox2, light grey arrow heads label RFP^{+} $Tuj1^{+}$ spinal cord/ET neurons, dark grey arrow heads mark RFP^{+} $Tuj1^{+}$ DRG neurons, and brown arrow heads point out RFP^{+} $GFAP^{+}$ glial cells. drg, dorsal root ganglion; et, ependymal tube; sc, spinal cord; sce, spinal cord ependyma. Bar = 50 μm.

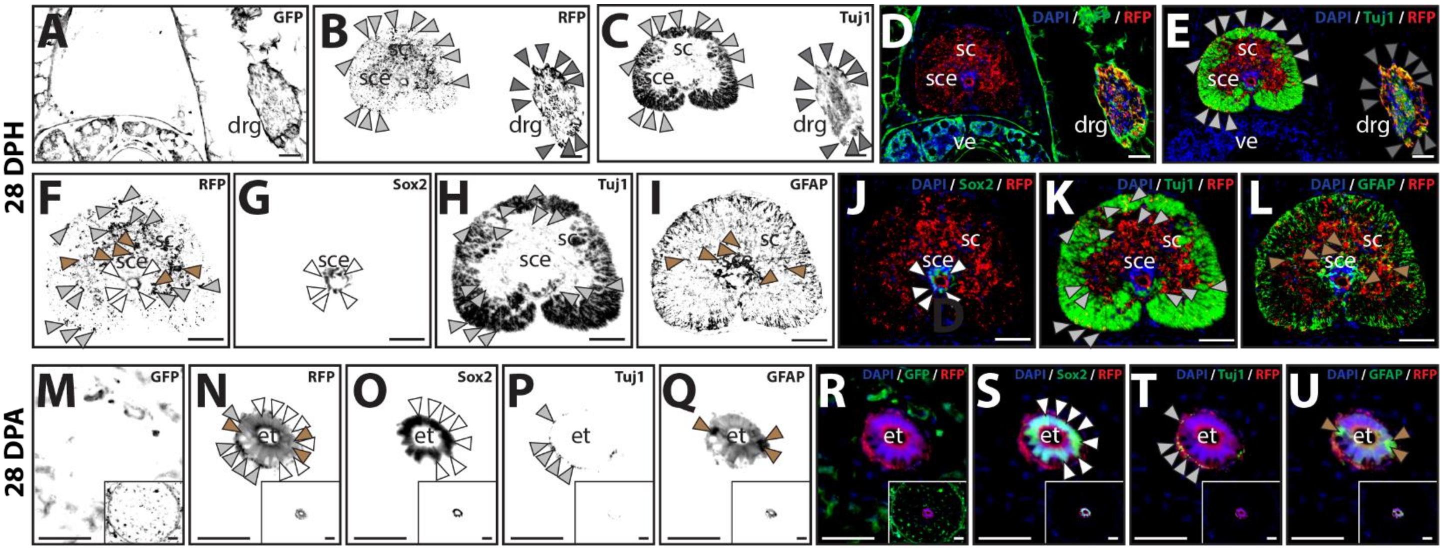

Supplementary Figure 11: Quantification of contribution of embryonic Sox2$^+$ NT cells pre-labeled with CreStoplight/GFP to Sox2$^+$ NSCs, Tuj1$^+$ neurons, and GFAP$^+$ glia in embryonic, adult, and regenerated tails. Lizard embryos (0 DPO) were transduced with Sox2-Cre and CreStoplight viral vectors in ovo and allowed to develop and hatch. Tail samples were collected from embryonic (7 DPO and 14 DPO), and adult lizards with original (28 DPH) or regenerated (28 DPA) tails. Cross sections of tail samples were analyzed by GFP, Sox2, Tuj1, and GFAP IF, and individual cells were identified with DAPI staining. Keyence microscope software was used to quantify the percentages of Sox2$^+$, Tuj1$^+$, and GFAP$^+$ cells that co-expressed RFP in sections taken along entire tail lengths. n=10 tails/animals for each time point/condition, and average percentages are presented below. Error bars mark standard deviations. Two-way ANOVA with pairwise Tukey's multiple comparison tests was used. Source data are provided as a Source Data file.

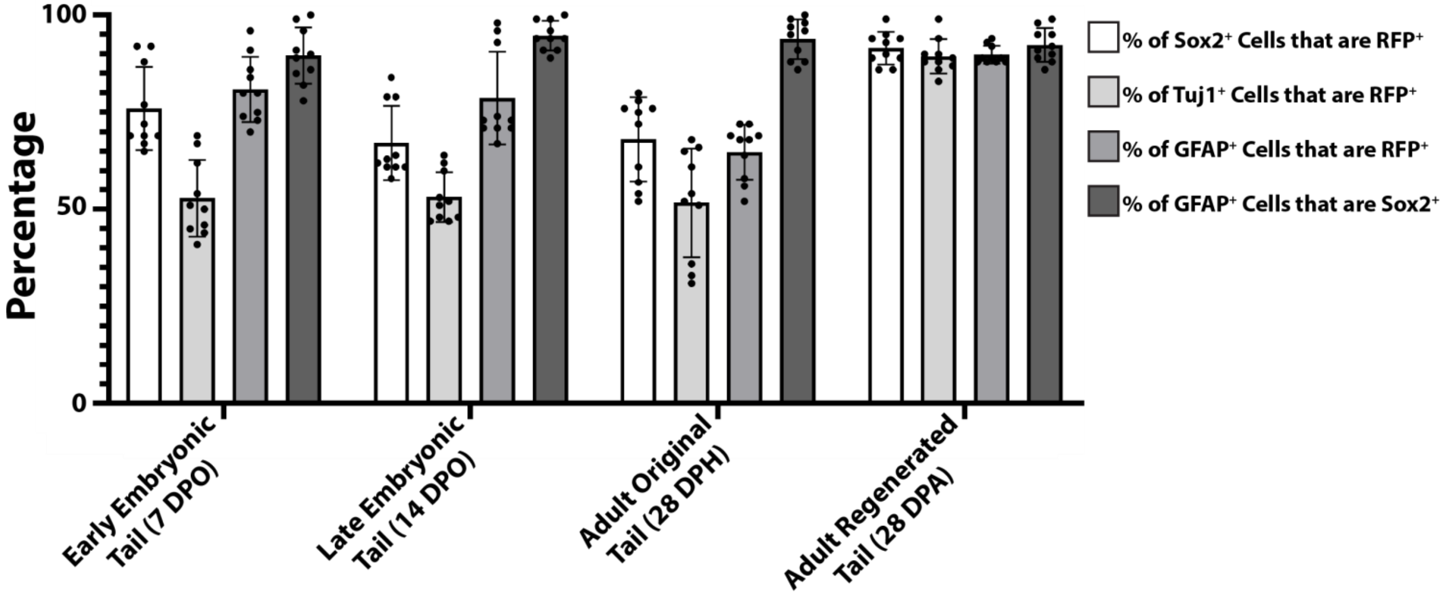

Supplementary Figure 12: Contributions of Sox2$^+$ embryonic NSCs to roof plate, lateral domains, and floor plate domains in embryonic, adult, and regenerated tails. Cross sections of **(Row A)** 7 DPO NTs, **(Row B)** 14 DPA NTs, **(Row C)** 28 DPH original tail spinal cord ependyma, and **(Row D)** 28 DPA regenerated tail ETs collected from lizards derived from embryos transduced with Sox2-Cre and CreStoplight constructs. Samples were analyzed for RFP signal and Pax7, Pax6, and Shh IF. For all rows, individual signal channels are presented to the left, and merged images of select channels are presented on the right. White arrow heads mark RFP$^+$ Pax7$^+$ roof plate regions, light grey arrow heads label RFP$^+$ Pax6$^+$ lateral domain, and dark grey arrow heads denote RFP$^+$ Shh$^+$ floor plate domains. cc, central canal; et, ependymal tube; nt, neural tube; sce, spinal cord ependyma. Bar = 50 µm.

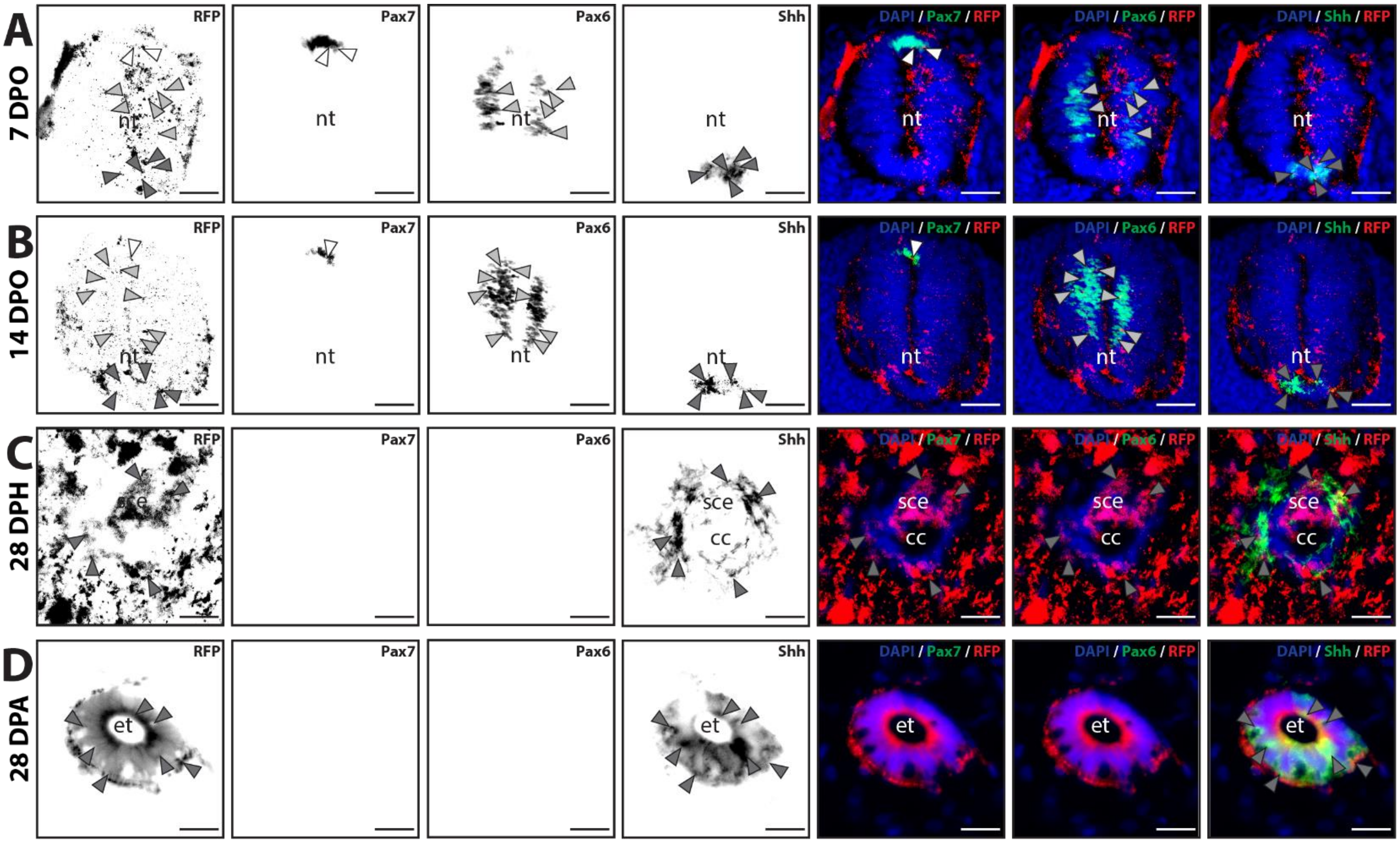

Supplementary Figure 13: Real time RT-PCR analyses of neurogenesis markers during embryonic and adult lizard tail neurosphere differentiation. NSCs isolated from embryonic NTs and original tail spinal cords were cultured as neurospheres for 3 passages. mRNA was isolated from either undifferentiated neurospheres or neurospheres cultured under differentiated conditions for 14 days and treated with either vehicle control, cyclopamine, or SAG. Expression of NSC/ependymal markers *Sox2* and *GFAP*, early neurogenesis markers *doublecortin* (*DCX*) and *NeuroD2*, and late differentiation marker *Tuj1* were analyzed by real time RT-PCR. Each experimental condition involved pooled mRNA from 10 neurospheres. n=3 experimental replicates. Results are presented as mean fold changes +/- SD compared to undifferentiated conditions. Two-way ANOVA with pairwise Tukey's multiple comparison tests was used. *, p<0.001 compared to Vehicle Control conditions. ns, not significant. Source data are provided as a Source Data file. Source data are provided as a Source Data file.

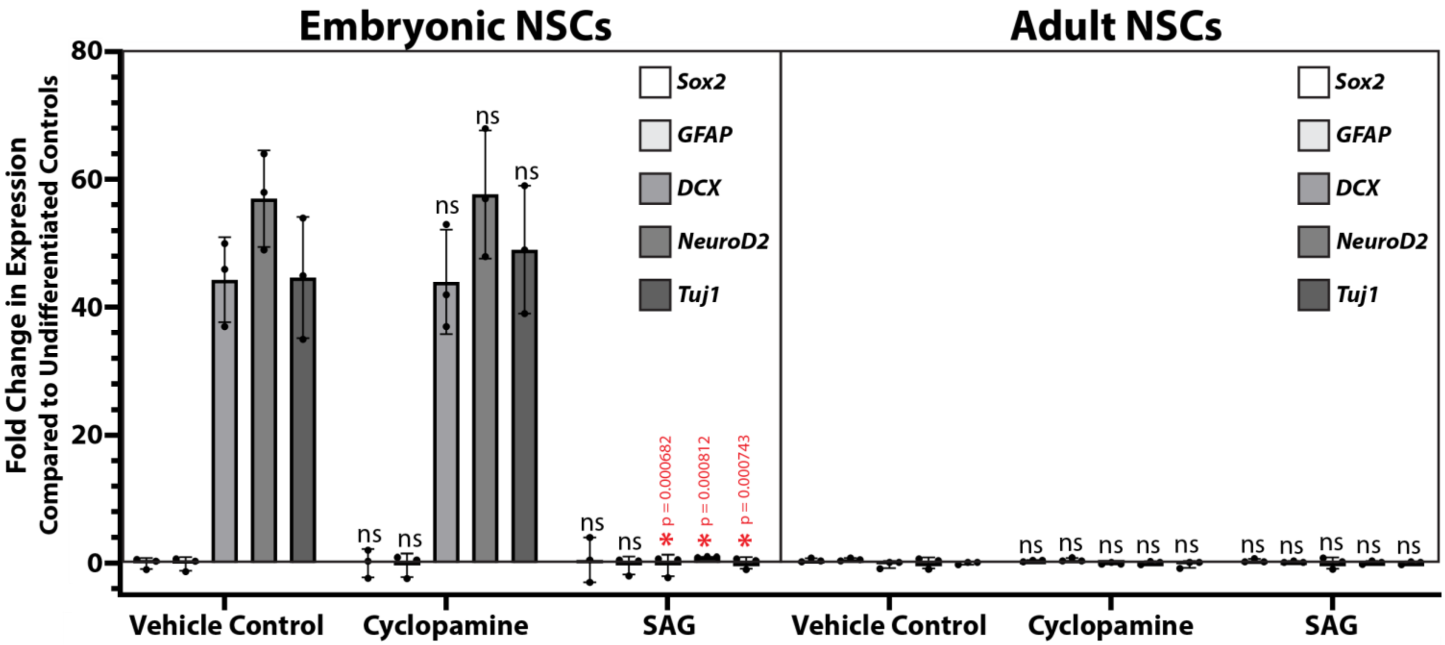

Supplementary Figure 14: Exogenous NSCs implanted into dorsal spinal cord ependyma populations reconstitute dorsal ETs upon tail amputation. (A) Experimental scheme for creating ET regions derived from exogenous NSC populations. NSCs were pre-labeled with fluorescent marker DiI before injection into dorsal regions of amputated tail ependyma. Following 28 days of tail regeneration, implanted DiI-labeled NSCs contribute to dorsal ET regions. **(B, C)** Fluorescent microscopy of a sagittally sectioned junction between original/regenerated tails 28 days after injection of DiI-labeled embryonic NSCs into dorsal regions of amputated tail spinal cord ependyma. Note reconstitution of dorsal, but not ventral, regenerated adult tail ET regions by exogenous embryonic NSCs. Asterix marks amputation site. Dashed line denotes initial amputation plane. et, ependymal tube; sce, spinal cord ependyma. Bar = 100 µm.

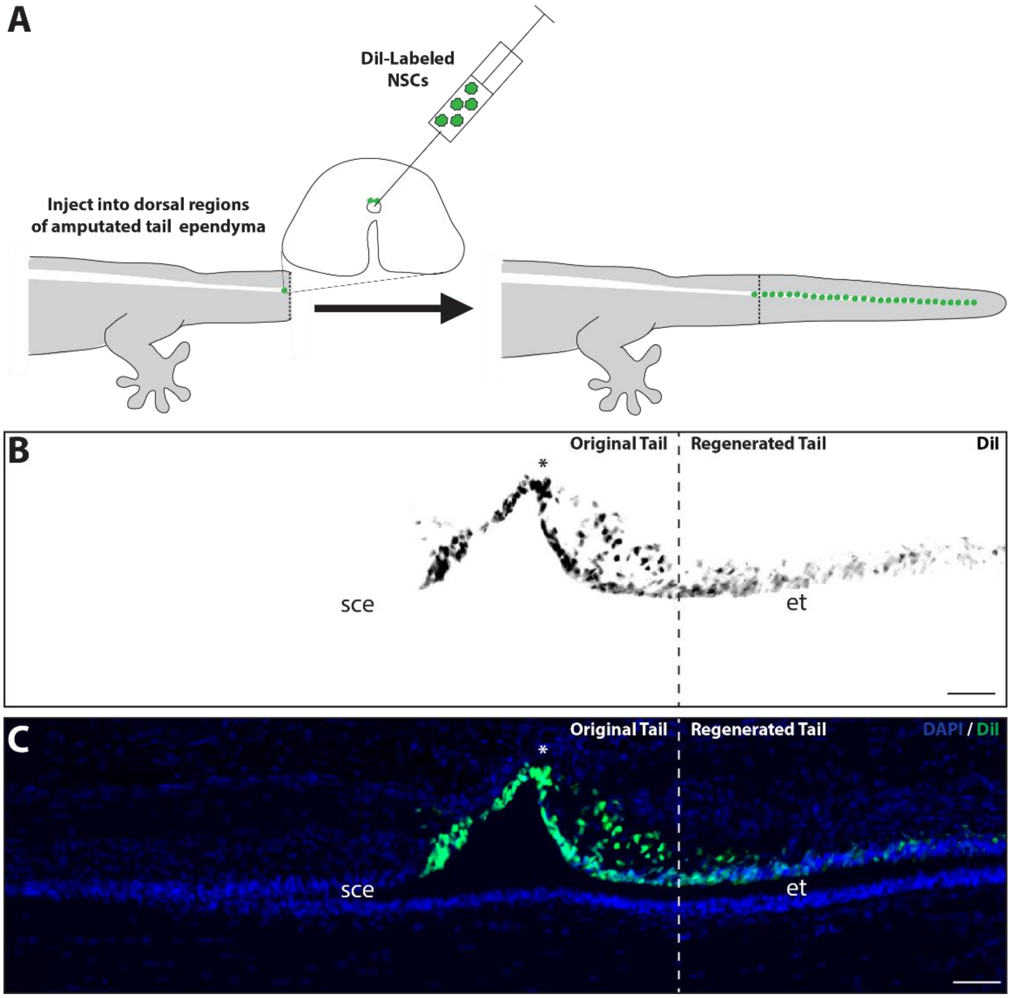

Supplementary Figure 15: FACS gating strategy used in Figure 8 B. Cells (Red) were single-gated from total events (Black) based on forward scatter (FSC) and side scatter (SSC). Double FSC AND SSC gates (blue) were used to select cells for GFP analysis. GFP^+ cells are colored green.

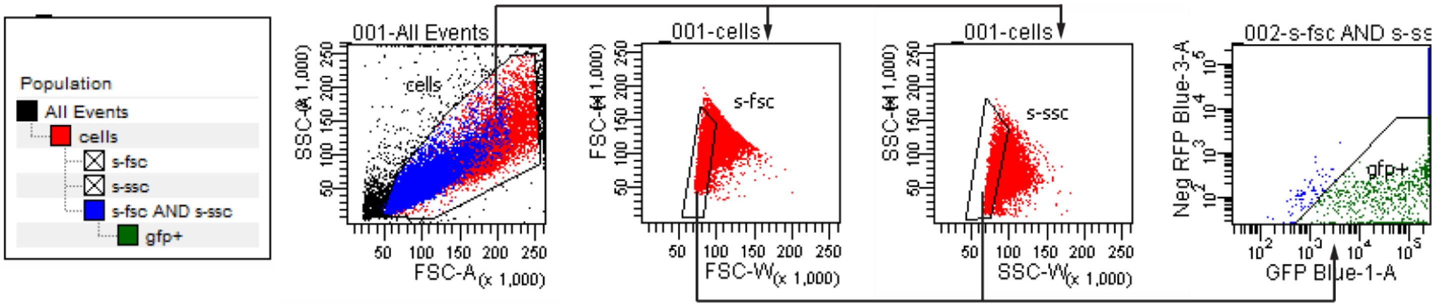

Supplementary Figure 16: Comparisons of *Smo* mRNA expression among *Smo*-CRISPR NSC clonal populations by real time RT-PCR. Embryonic NSCs were electroporated with complexes of Cas9/*LlSmo*-gRNA/GFP HDR template and clonally expanded to yield 50 *Smo*-CRISPR clonal populations. Control-CRISPR NSCs were treated with nonbinding 'scramble' gRNA rather than *LlSmo*-gRNA. Samples of each *Smo*-CRISPR clonal population and Control-CRISPR cells were analyzed by real time RT-PCR for *Smo* mRNA expression. Expression levels of *Smo*-CRIPSR cell populations were normalized to those of Control-CRISPR and are presented in the histograms below as mean values +/- SD. Two-way ANOVA with pairwise Tukey's multiple comparison tests was used. *, p<0.01 compared to Control-CRSIPR NSC *Smo* mRNA expression levels. n=5 for all measurements. Source data are provided as a Source Data file.

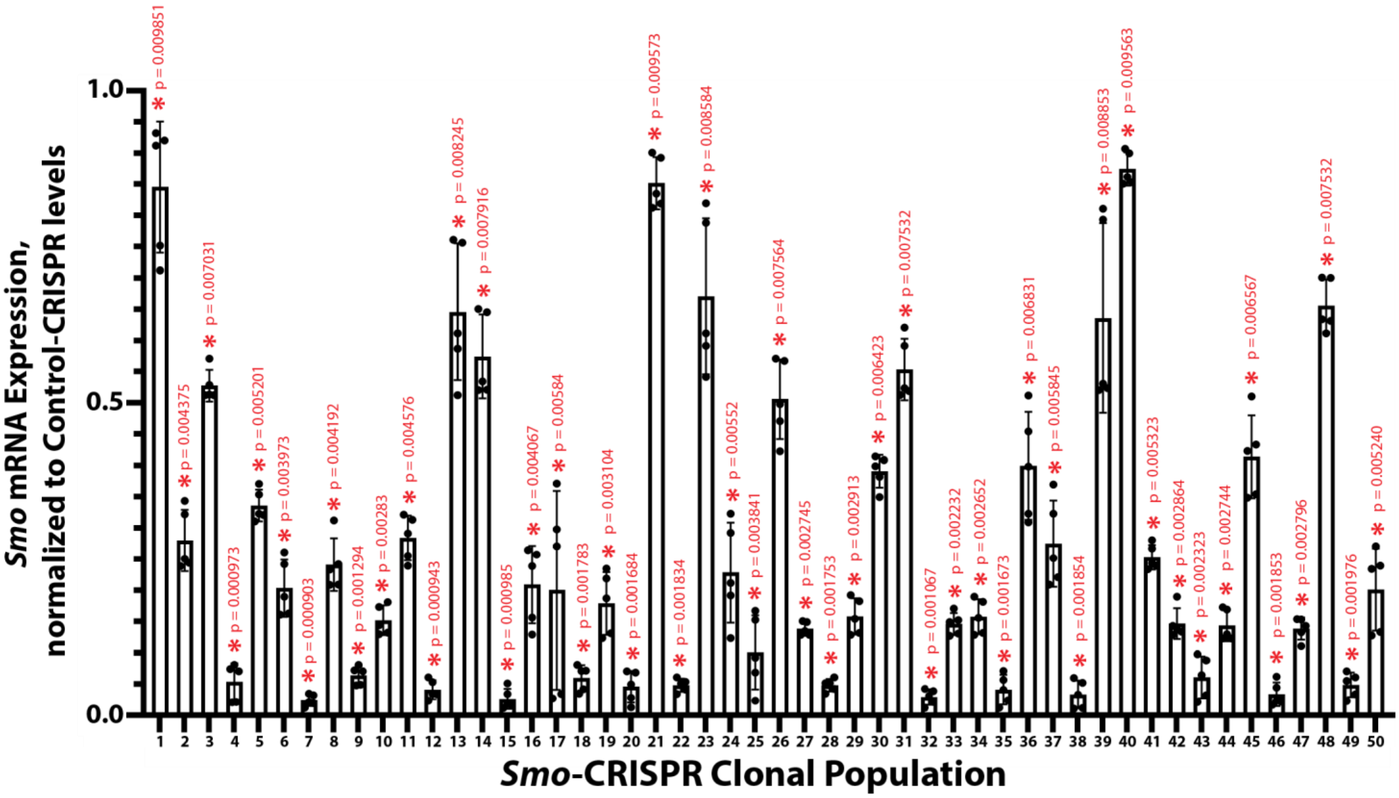

Supplementary Figure 17: Effects of SAG stimulation on *Gli1* mRNA expression in *Smo*-CRISPR NSC clonal populations. Clonal populations derived from *Smo*-CRISPR NSCs transfected with Cas9/*LlSmo*-gRNA/GFP HDR plasmid were treated with 100 nM SAG or vehicle control for 48 hours. Samples of each *Smo*-CRISPR population were analyzed by real time RT-PCR for *Gli1* mRNA expression. Expression levels of SAG-stimulated populations were normalized to those of control conditions and are presented below as mean values +/- SD. Control-CRISPR NSCs included as positive controls exhibited increases in *Gli1* expression in response to SAG treatment, while several *Smo*-CRIPSR populations did not respond to hedgehog stimulation. Two-way ANOVA with pairwise Tukey's multiple comparison tests was used. *, p<0.01 compared to fold changes in Control-CRSIPR NSC Gli1 mRNA expression levels. n=5 for all measurements. Source data are provided as a Source Data file.

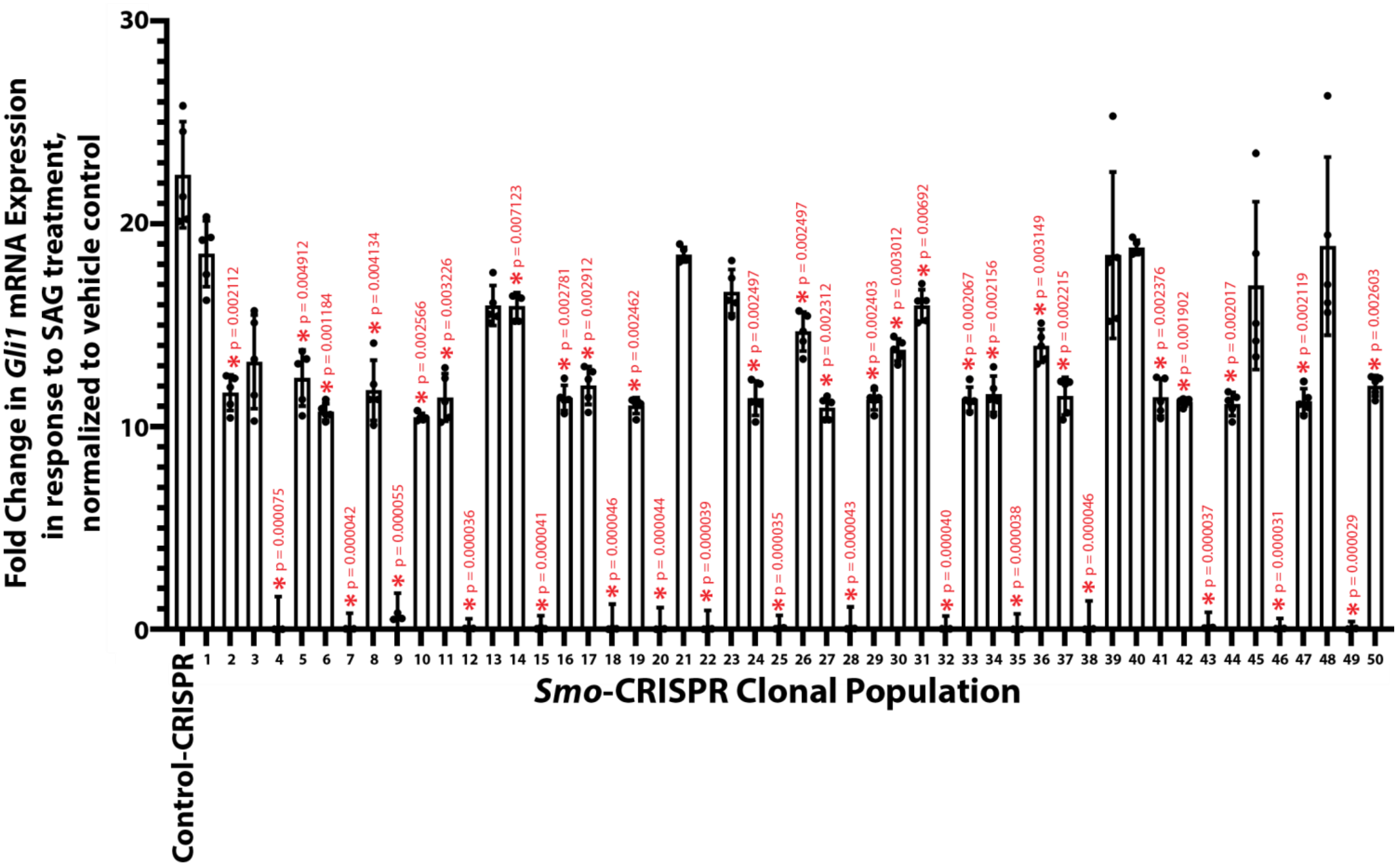

Supplementary Figure 18: Comparisons of *Gli1* promoter responsiveness to SAG stimulation in *Smo*-CRISPR NSC clonal populations. *Smo*-CRISPR clonal NSC populations were co-transfected with 1) a hedgehog pathway reporter construct involving *Gli1* promoter-driven expression of firefly luciferase (*Gli1*-FLuc) and 2) an internal control plasmid involving constitutively active promoter CMV-driven expression of Renilla luciferase (RL-CMV). Transfected cells were stimulated with 100 nM SAG or vehicle control for 2 hours before incubation with Firefly and Rinella luciferase substrates suitable for use with live cells. Firefly and Rinella luminescences were measured for each *Smo*-CRISPR clonal population. Firefly luminescences were normalized to corresponding Rinella measurements and are presented below as mean values +/- SD. (n=5). Control-CRISPR included as positive controls exhibited increased *Gli1* promoter activity in response to SAG treatment, while *Gli1* promoters of several *Smo*-CRISPR populations were unresponsive to SAG treatment. Two-way ANOVA with pairwise Tukey's multiple comparison tests was used. *, p<0.001 compared to Control-CRSIPR NSC *Gli1* promoter luciferase activity. n=5 for all measurements. Source data are provided as a Source Data file.

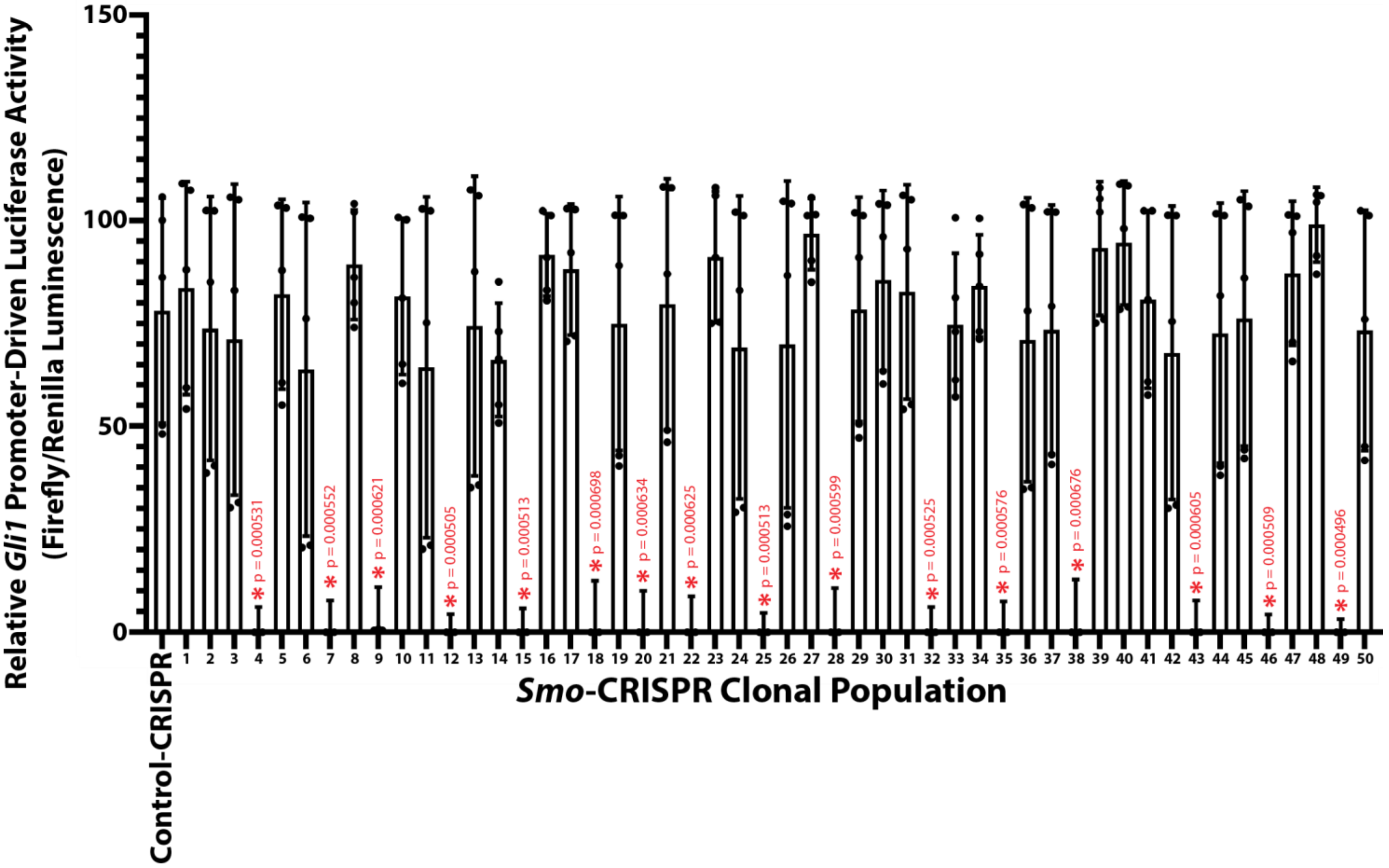

Supplementary Figure 19: Contribution of exogenous embryonic NSCs to dorsal and ventral ET regions. *Smo* KO NSCs labeled with GFP and control NSCs pre-labeled with DiI were engrafted to dorsal ependyma of amputated tail spinal cords. Following 28 days of regrowth, regenerated tails were collected and processed for histology. ET cross sections were analyzed by IF/fluorescence microscopy for GFP/DiI signal and Sox2 expression every 1 mm along entire tail lengths. Horizontal lines drawn through the centers of ETs bisected tube images into dorsal and ventral regions. Dorsal and ventral Sox2 and GFP/DiI signal areas were quantified for each cross-section along tail lengths. n=10 different animals/tails per condition. Percentages of $Sox2^+$ IF signal areas co-expressing GFP/DiI were calculated for dorsal and ventral ET regions and are presented in the histograms below as mean values +/- SD. Two-way ANOVA with pairwise Tukey's multiple comparison tests was used. Dorsal ET regions exhibited significantly higher GFP/DiI signal from labeled exogenous NSC populations than ventral regions, ($p<0.001$ for all positions and conditions), indicating that initial NSC spatial arrangements were maintained along regenerated ET lengths. Source data are provided as a Source Data file.

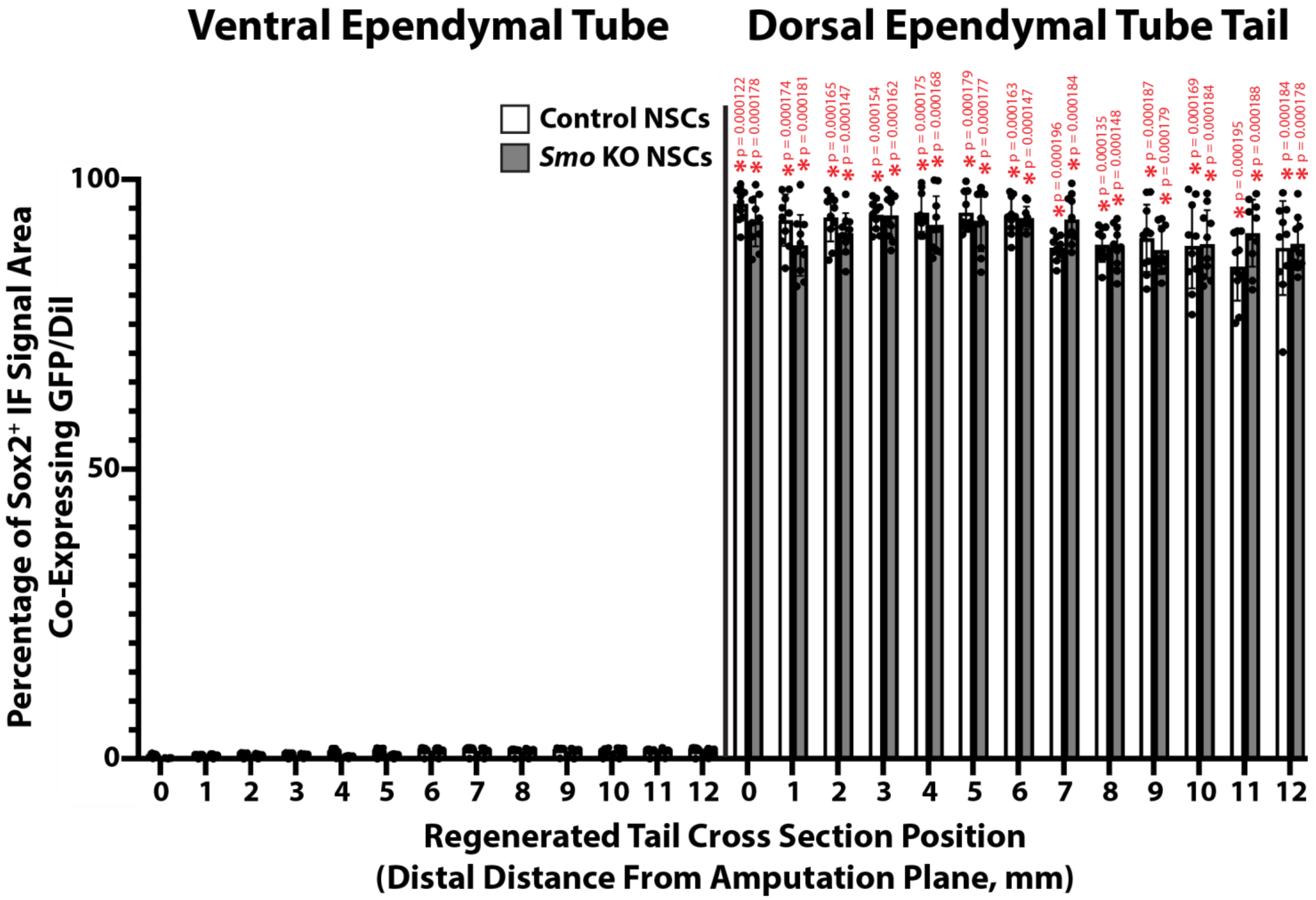

Supplementary Figure 20: Neural differentiation of exogenous embryonic NSCs during incorporation in adult regenerated tails. GFP-labeled *Smo* KO NSCs and control NSCs pre-labeled with DiI engrafted to dorsal spinal cord ependyma cell populations. Following 28 days of regrowth, regenerated tails were collected and processed for histology. ET cross sections were analyzed by IF/fluorescence microscopy for GFP/DiI signal and Tuj1 expression every 1 mm along entire tail lengths. n=10 different animals/tails per condition. Tuj1 and GFP/DiI signal areas were quantified for each cross-section, and percentages of $Tuj1^+$ IF signal areas co-expressing GFP/DiI are presented in the histograms below as mean values +/- SD. $Tuj1^+$ neural cells co-expressed GFP in lizards treated with *Smo* KO NSCs, indicating significantly higher levels of neurogenesis in *Smo* KO NSCs than control cells. Two-way ANOVA with pairwise Tukey's multiple comparison tests was used. *, p<0.001 compared to corresponding Control NSC condition for each measurement. Source data are provided as a Source Data file.

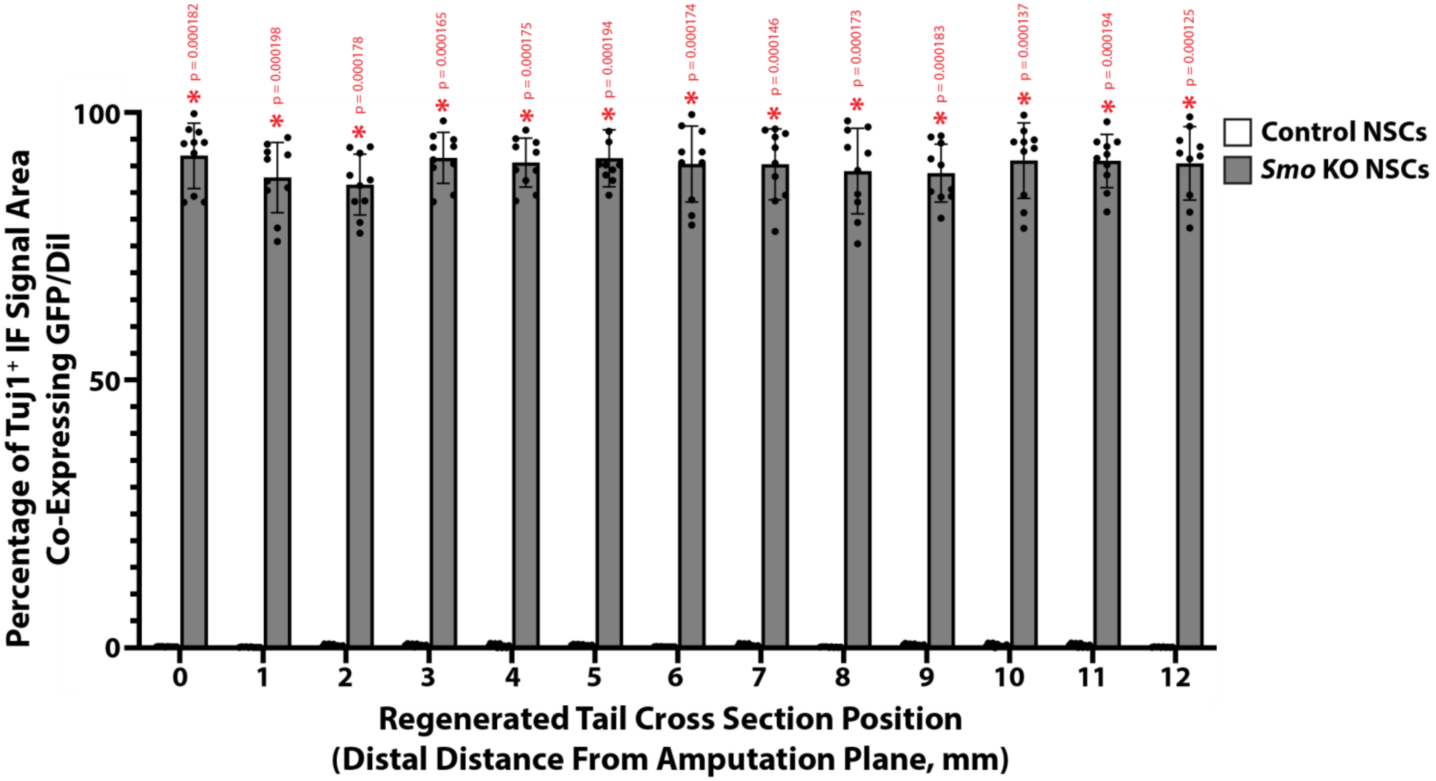

Supplementary Figure 21: ***L. lugubris*** **karyotype (2n=22).**

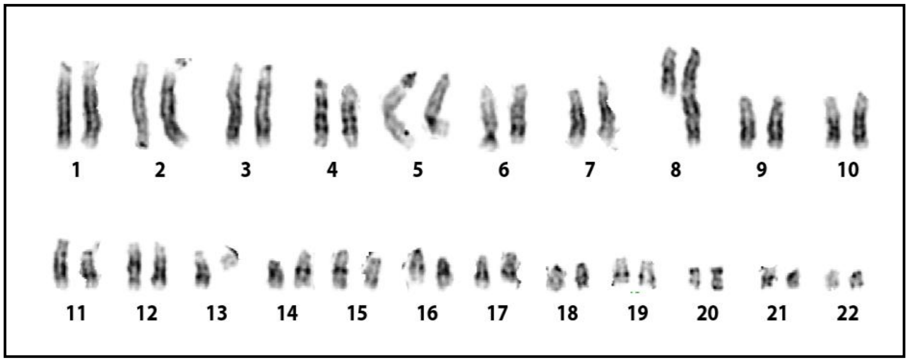

Supplementary Table 1: Promoter, gene, and gRNA sequences used for work with the mourning gecko (*L. lugubris*).

Name	Sequence
Sox2 promoter (GenBank Accession Number MZ983792)	GTAGGGTGTTCTTTCAGGCGAGATTCTCTGGTTTGGAGTAGGCTGAAGAGGAC<br>GGAGGCGATCAGATGTTTGGCTGGGGGAGGGGAACTCGAGAGGCCAGCTCGG<br>CGTGGTTGTCAGGCGGGCAAGTGGGGGGGGGAGGGGCGCCAACAGCCTAGA<br>GGGAACTTGGCGTCGGTCAGCCAAAGAAAGCAGAAGCCATCTCCTCGTTACT<br>CGGAAGTAAACCCCGTGGGTCACAAGGGGACAGACGACTTTAGGGCTGCCAC<br>ATAAGGCCTATTTGCATGGGTTGCAGCTTGAATCCTGAATAGCGTTGCTGAAT<br>CCCCTCTACGGAGCCTGCCTTCAAGTCATTGCGCCCAGGATCGCAGGCTTGGA<br>GGTCAACAGGGCGAAGCAGGCCCCGTTCTGGCGCTCAAGTGGCTTGCGGACC<br>GCGCTTTCCCCGCCCTGCCCTTCGCGCTTTCCCCGCCCTGCTTGGCTCCTCCGC<br>ACAGTAATCTGTTGGGAAGGCAGGGCCCCCGTGTGCCACAAGCCTCGTTGCC<br>AGGTGAACAGAGGTGCGGAGCCAGCAGGGGCTGAGGGAAACCGTAGTCGCC<br>TGACGACTGGGTGACTAGGAAATCTTGCGGATCGAGGCCTGAGGGAGGGCCC<br>TTCCGGGCTTCCCGTGCAGAGATCTTTCTGCGGAAGGAACTATTATGAAACGA<br>TTTCGAGCAGCACACTGGAGTGGGGGAGAGGGAGACAAGACTCTGCCCTCCT<br>TGGATTCGTGGCGGTTTGCGCTCCGGGAGGATAGGAAAGAGGCGCCACCGGA<br>GAAACCCATCGAGGCCGCTGGAAAAGGAAGTGAGACTGGAGCAGATCTCCCC<br>GCCTGCCGTCGAGTCCCCGAAAAAGAAGCCGTCAGAAGAGCGGGGGGGGGG<br>TTCCTCTCCTCTCTCTCTCTCTTCCAGCCCCTCTCTTGGGGGGGGGAACTGGCA<br>CCCCCCCCCATTCACCTTAAGTCAACCTCAGGCCGTGGCAAGAGCGAGTCTTT<br>TTTTCA
LlSmo Exon 2 (GenBank Accession Number MZ983791)	CTGTGTGCCGTNTTCTACNTGCCCAAGTGTGAGGANGGCATGGTGGAGCTGCC<br>CAGCCAGACCCTGTGCCAGGCGACCCGGGGGCCCTGCACCATCGTGGAACGG<br>GAACGGGGCTGGCCCGATTTCCTGAAGTGCACCACAGACCGCTTCCCGGAAG<br>GGTGCCCGAATGAGGTCCAGAATATCAAGTTCAACAGCTCGGGGCAGTGCGA<br>GGCCCCCCTGGTGCGGACCGACAACCCCAAGAGCTGGTACGAGGACGTCGAG<br>GGCTGCGGGATCCAGTGCCAGAACCCCCTTTTCACCGAGAAGGAGCACCGCG<br>AAATGCACGTCTACATCGCTGCCTTCAGCTTGGTCACCATCATCTGCACCTTCT<br>TTACCTTGGCCACCTTCGTAGCCGACTGGAAGAACTCGAACCGCTACCCGGCC<br>GTCATCCTCTTCTACGTCAACGCCTGCTTATTCATGGGCAGCATCGGGTGGCT<br>GGCGCAGTTCATGGACGGGGCCCGCAGCGAGATCGTGTGCCGCGCGGACGGG<br>ACCATGCGCCTGGGAGAACCGACCTCCAATGAGACCCTCTCGTGCGTGATCAT<br>CTTTGTCATCGTCTACTACTCCCTGATGTCGGGCGTGATCTGGTTCGTCATGCT<br>GACCTACGCCTGGCACACCTCCTTCAAGGCCCTGGGCACCACCTACCAGCCGC<br>TGCTGGGCAAGACCTCCTACTTCCACCTCGTGACCTGGTCGATTCCGTTCGTC<br>CTCACAGTCACCATTCTGGCGGTCGCGCAGGTGGATGGAGACTCGGTCAGCG<br>GCATCTGCTTCGTGGGGTACAAGAACTACCACTATCGGGCGGGTTTCGTGCTG<br>GCCCCCATCGGCCTGGTGTTGATCGTGGGGGGCTATTTCCTGANCCN
LlSmo-gRNA	GGCCCTGCACCATCGTGGAA
Control Scramble gRNA	GCACTACCAGAGCTAACTCA
Smo real time RT-PCR forward	TACATCGCTGCCTTCAGCTT
Smo real time RT-PCR reverse	ACTGTGAGGACGAACGGAAT
GLI1 real time RT-PCR forward	CCTACGTCTGCAAGATCCC
GLI1 real time RT-PCR reverse	CCTGCCCTCACTATCCCTCT
GAPDH real time RT-PCR forward	CCATGTTTGTGATGGGTGTC
GAPDH real time RT-PCR reverse	CTTCTGTGTGGCTGTGATGG

Supplementary References

1 Yang, Y. S. & Hughes, T. E. Cre stoplight: a red/green fluorescent reporter of Cre recombinase expression in living cells. *Biotechniques* **31**, 1036, 1038, 1040-1031 (2001).
